# The Effects of *Syzygium samarangense*, *Passiflora edulis* and *Solanum muricatum* on Alcohol-Induced Liver Injury

**DOI:** 10.3390/ijms17101616

**Published:** 2016-09-26

**Authors:** Yu-Jie Zhang, Tong Zhou, Fang Wang, Yue Zhou, Ya Li, Jiao-Jiao Zhang, Jie Zheng, Dong-Ping Xu, Hua-Bin Li

**Affiliations:** 1 Guangdong Provincial Key Laboratory of Food, Nutrition and Health, School of Public Health, Sun Yat-sen University, Guangzhou 510080, China; zhyujie3@mail2.sysu.edu.cn (Y.-J.Z.); zhout43@mail2.sysu.edu.cn (T.Z.); missingfeng@yeah.net (F.W.); zhouyue3@mail2.sysu.edu.cn (Y.Z.); liya28@mail2.sysu.edu.cn (Y.L.); zhangjj46@mail2.sysu.edu.cn (J.-J.Z.); zhengj37@mail2.sysu.edu.cn (J.Z.); xudp@mail2.sysu.edu.cn (D.-P.X.); 2 South China Sea Bioresource Exploitation and Utilization Collaborative Innovation Center, Sun Yat-sen University, Guangzhou 510006, China

**Keywords:** alcohol, liver injury, *Syzygium samarangense*, *Passiflora edulis*, *Solanum muricatum*

## Abstract

Previous studies have shown that fruits have different effects on alcohol metabolism and alcohol-induced liver injury. The present work selected three fruits and aimed at studying the effects of *Syzygium samarangense*, *Passiflora edulis* and *Solanum muricatum* on alcohol-induced liver injury in mice. The animals were treated daily with alcohol and fruit juices for fifteen days. Chronic treatment with alcohol increased the levels of aspartate transaminase (AST), alanine transaminase (ALT), total bilirubin (TBIL), triglyceride (TG), malondialdehyde (MDA), and decreased total protein (TP). Histopathological evaluation also showed that ethanol induced extensive fat droplets in hepatocyte cytoplasm. *Syzygium samarangense* and *Passiflora edulis* normalized various biochemical parameters. *Solanum muricatum* increased the level of ALT and induced infiltration of inflammatory cells in the liver. These results strongly suggest that treatment with *Syzygium samarangense* and *Passiflora edulis* could protect liver from the injury of alcohol, while *Solanum muricatum* could aggravate the damage.

## 1. Introduction

Alcohol consumption has been commonplace since ancient times all over the world [1]. Long-term excessive alcohol consumption can result in several diseases, such as gastrointestinal injury, alcoholic hepatic disease (including cirrhosis), pancreatitis, hepatocarcinoma, esophagus cancer, breast cancer, hypertension, and strokes [2,3]. Alcohol-related disease is a considerable cause of morbidity and mortality, and affects millions of individuals worldwide.

After drinking, only a small amount of alcohol may be oxidized in the stomach by alcohol dehydrogenase (ADH) isoforms, and most alcohol enters the systemic circulation by the small intestine through passive diffusion [4]. About 90% of ethanol is metabolized in liver, so liver is the most adversely influenced organ [5]. Ethanol is metabolized into acetaldehyde by alcohol metabolizing enzymes, including ADH (present in cytosol), cytochrome P450 2E1 (CYP2E1) (present in microsomes), and catalase (present in peroxisomes) [6,7]. The acetaldehyde is further oxidized into acetic acid by aldehyde dehydrogenase (ALDH) in the mitochondria. The CYP2E1-mediated metabolism of ethanol requires nicotinamide adenine dinucleotide phosphate (NADPH) and the incomplete reduction of oxygen, which results in the generation of free radical species [8,9]. Chronic alcohol consumption is able to increase the activity of CYP2E1 dramatically, which generates abundant oxidants, such as reactive oxygen species (ROS) and free radicals in the liver [10,11,12].

Oxidants can induce the peroxidation of polyunsaturated fatty acid side chains of membrane phospholipids and lipoproteins. The antioxidant defense system can protect cells from different species of oxidants. Antioxidant enzymes and non-enzymatic antioxidants form the antioxidant defense system. The antioxidant enzymes in vivo include superoxide dismutase (SOD), glutathione peroxidase (GPx), catalase (CAT), and hemeoxygenase, and the non-enzymatic antioxidants in vivo contain glutathione (GSH), metallothioneins (MTs), vitamins, ubiquinone, and uric acid [13]. Consumption of alcoholic beverages may lead to the depletion of endogenous antioxidants in the liver. Both the depletion of the endogenous antioxidants and the accumulation of oxidants can induce oxidative stress. Accordingly, oxidative stress is thought to be a key factor in the pathogenesis of alcohol-induced liver injury.

Alcohol is sometimes consumed with fruit. Several fruits have been reported to have a protective effect on alcohol-induced liver injury, which may be an alternative to synthetic medicines in the prevention of alcohol-induced liver injury and dysfunction [14,15]. However, some fruits have been shown to aggravate the adverse effects induced by alcohol [16]. According to previous studies, three fruits—*Syzygium samarangense*, *Passiflora edulis* and *Solanum muricatum*—may affect alcohol metabolism [17]. Therefore, these fruits were selected to evaluate their effects on chronic alcohol-induced liver injury.

## 2. Results and Discussion

### 2.1. The Effects of Syzygium samarangense, Passiflora edulis and Solanum muricatum on the Levels of Aspartate Transaminase (AST), Alanine Transaminase (ALT) and Alkaline Phosphatase (ALP) in Serum

**Figure 1:**
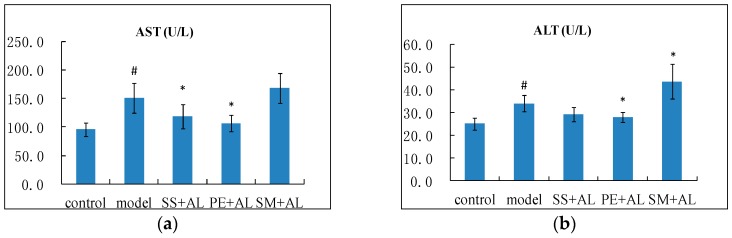

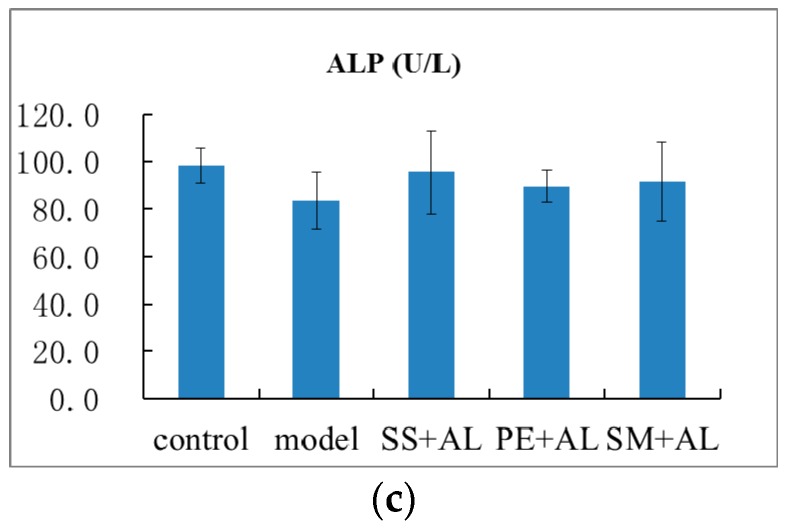
Effects of *Syzygium samarangense*, *Passiflora edulis* and *Solanum muricatum* on the levels of aspartate transaminase (AST) (**a**); alanine transaminase (ALT) (**b**); and alkaline phosphatase (ALP) (**c**) in serum. ^#^ indicates that there is a significant (*p* < 0.05) difference between the model group and the control group. * indicates that there is a significant (*p* < 0.05) difference between the treatment group and the model group. SS + AL: *Syzygium samarangense* + alcohol; PE + AL: *Passiflora edulis* + alcohol; SM + AL: *Solanum muricatum* + alcohol.

As shown in Figure 1, ethanol significantly (*p* < 0.05) raised the levels of ALT and AST in the serum of the model group compared with the control group, which indicated the occurrence of liver injury. The levels of ALP were not affected by alcohol and fruit. The levels of AST markedly decreased (*p* < 0.05) from treatment with *Syzygium samarangense* and *Passiflora edulis*. The serum ALT level significantly decreased (*p* < 0.05) by treatment with *Passiflora edulis*, but significantly (*p* < 0.05) increased in the treatment group with *Solanum muricatum* juice.

Long-term consumption of excess alcohol can lead to liver injury [18]. The activities of serum ALT and AST are liver injury biomarkers, and their significant elevations denote increased permeability and injury, necrosis of hepatocytes, or a combination of the above [19]. In this study, chronic ingestion of alcohol resulted in increased levels of ALT and AST, which reflected alcohol-induced liver injury. Treatment with *Syzygium samarangense* and *Passiflora edulis* normalized these indexes, which indicated that they reduced the liver damage. However, treatment with *Solanum muricatum* increased the level of ALT in alcoholic mice, which indicated that it could aggravate alcohol-induced liver injury.

### 2.2. The Effects of Syzygium samarangense, Passiflora edulis, and Solanum muricatum on the Levels of Serum Triglyceride (TG), Total Protein (TP), Total Bilirubin (TBIL), and Hepatic TG

**Figure 2:**
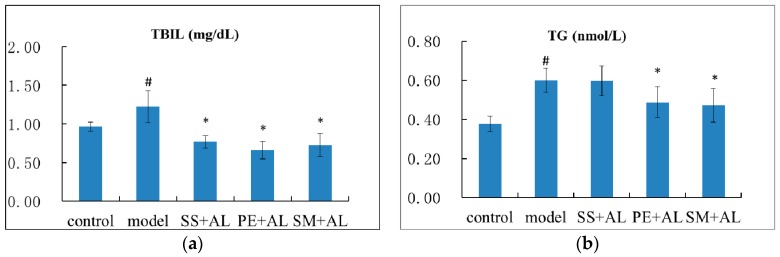

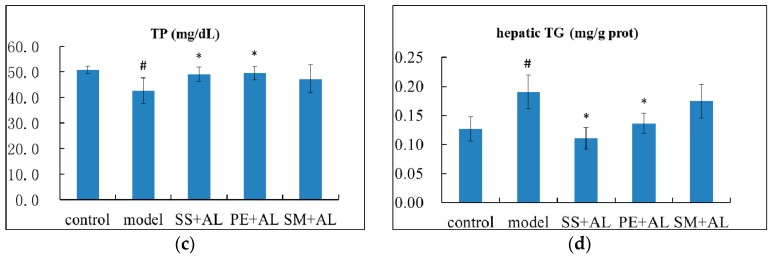
Effects of *Syzygium samarangense*, *Passiflora edulis* and *Solanum muricatum* on the levels of serum total bilirubin (TBIL) (**a**); triglyceride (TG) (**b**); total protein (TP) (**c**); and hepatic TG (**d**). ^#^ indicates that there is a significant (*p* < 0.05) difference between the model group and the control group. * indicates that there is a significant (*p* < 0.05) difference between the treatment group and the model group. SS + AL: *Syzygium samarangense* + alcohol; PE + AL: *Passiflora edulis* + alcohol; SM + AL: *Solanum muricatum* + alcohol.

Another hallmark of the chronic alcohol-induced liver injury was indicated by the elevated levels of serum TBIL, TG, and hepatic TG, as well as the decreased serum TP content. As shown in Figure 2, treatment with *Syzygium samarangense*, *Passiflora edulis* and *Solanum muricatum* juices alleviated the boosted level of TBIL. Treatment with *Passiflora edulis* significantly (*p* < 0.05) decreased the level of serum TG and hepatic TG, as well as significantly (*p* < 0.05) increased TP content. *Syzygium samarangense* treatment significantly (*p* < 0.05) increased TP content and significantly (*p* < 0.05) decreased hepatic TG content. *Solanum muricatum* significantly (*p* < 0.05) decreased the level of serum TG, but not the level of serum TP and hepatic TG.

Bilirubin is an endogenous organic anion that binds reversibly to albumin. When transported to liver, it is conjugated with glucuronic acid and excreted in the bile. Increased content of bilirubin in serum reflects the functional lesion of the liver [20]. Treatment with *Syzygium samarangense* and *Passiflora edulis* juices alleviated the boosted level of TBIL, which indicated that they could restore the damaged liver function.

Alcohol can alter the fatty acid composition in the liver. During alcohol metabolism, an increased ratio of reduced nicotinamide adenine dinucleotide to oxidized nicotinamide adenine dinucleotide could impair fatty acid β-oxidation and the tricarboxylic acid cycle in hepatocytes. Alcohol consumption can lead to severe free fatty acid overload, increased TG synthesis, and subsequent steatosis [21]. Based on the above results, *Syzygium samarangense* and *Passiflora edulis* treatment decreased hepatic TG content, which indicated that they could improve hepatocyte steatosis.

### 2.3. The Effects of Syzygium samarangense, Passiflora edulis and Solanum muricatum on the Contents of Malondialdehyde (MDA) and Glutathione (GSH) in the Liver

**Figure 3:**
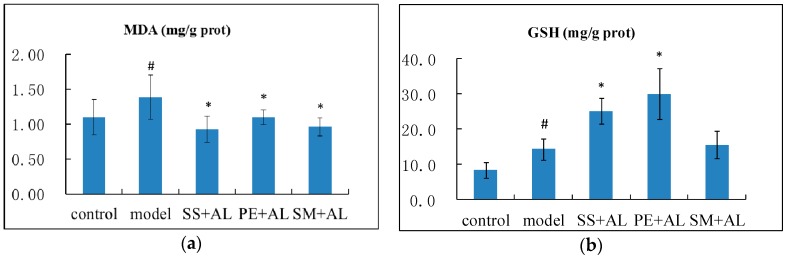
Effects of *Syzygium samarangense*, *Passiflora edulis* and *Solanum muricatum* on the contents of MDA (**a**); and GSH (**b**) in the liver. ^#^ indicates that there is a significant (*p* < 0.05) difference between the model group and the control group. * indicates that there is a significant (*p* < 0.05) difference between the treatment group and the model group. SS + AL: *Syzygium samarangense* + alcohol; PE + AL: *Passiflora edulis* + alcohol; SM + AL: *Solanum muricatum* + alcohol.

Malondialdehyde (MDA) is one of the major end products of lipid peroxidation by ROS and can directly indicate the damage of ROS on liver. Thus, MDA is more often used than ROS in the literature [22,23]. Chronic alcohol intake significantly (*p* < 0.05) increased the level of MDA in the liver (Figure 3), and treatment with *Syzygium samarangense*, *Passiflora edulis* and *Solanum muricatum* juices significantly (*p* < 0.05) decreased it.

GSH is an antioxidant that can protect cells from free radicals and peroxides. Chronic alcohol intake significantly (*p* < 0.05) increased the level of GSH in the liver, and treatment with *Syzygium samarangense* and *Passiflora edulis* juices increased it further.

Oxidative stress is thought to be a key factor in the pathogenesis of alcohol induced liver injury. Excessive ROS and free radicals could result in lipid peroxidation of hepatocytes, which is thought to be a significant mechanism associated with liver injury after long-term alcohol consumption [24]. MDA is the product of lipid peroxidation and increases in an alcohol-damaged liver. In the present study, alcohol increased the content of MDA, which is similar to a previous study [25] indicating an increased peroxidation and failure of the antioxidant defense system. Treatment with *Syzygium samarangense*, *Passiflora edulis* and *Solanum muricatum* restored the content of MDA back to normal, which showed that they can decrease oxidative stress induced by alcohol.

GSH is the most abundant thiol in mammals, and its major function is detoxification and protection against oxidants [26]. There are studies showing that chronic ethanol intake can result in an increase in GSH in human hepatoma cell line VL-17A [27]. Similarly, in this study, the content of GSH was stimulated in the model group with respect to the control group. Treatment with *Syzygium samarangense* and *Passiflora edulis* increased the content of GSH further in alcoholic mice, which might protect the liver from the damage of alcohol.

### 2.4. The Effects of Syzygium samarangense, Passiflora edulis and Solanum muricatum on the Levels of Hepatic Superoxide Dismutase (SOD) and Catalase (CAT)

**Figure 4:**
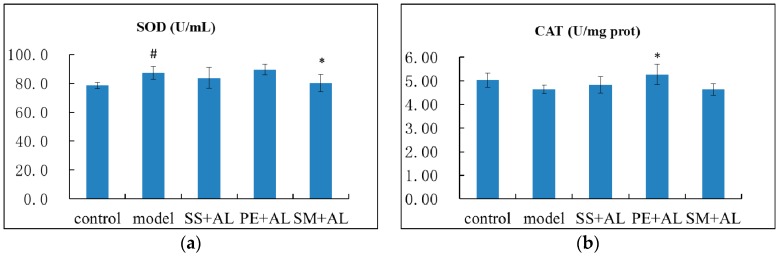
Effects of *Syzygium samarangense*, *Passiflora edulis* and *Solanum muricatum* on the levels of hepatic SOD (**a**); and CAT (**b**). ^#^ indicates that there is a significant (*p* < 0.05) difference between the model group and the control group. * indicates that there is a significant (*p* < 0.05) difference between the treatment group and the model group. SS + AL: *Syzygium samarangense* + alcohol; PE + AL: *Passiflora edulis* + alcohol; SM + AL: *Solanum muricatum* + alcohol.

The antioxidant enzymes, including CAT and SOD, represent the defense response to excessive free radicals. In the present study, the activity of hepatic SOD was increased significantly (*p* < 0.05) in alcoholic mice, and treatment with *Solanum muricatum* juice significantly (*p* < 0.05) brought it down (Figure 4). The activity of hepatic CAT was decreased in alcoholic mice (without significance, *p* > 0.05), but treatment with *Passiflora edulis* juice significantly (*p* < 0.05) raised it.

Antioxidant enzymes protect the liver from free radicals. It is believed that SOD and CAT play important roles in the enzymatic defense system. CAT is a classical oxidative biomarker, which mainly exists in peroxisomes of aerobic cells. SOD (a metalloenzyme) converts superoxide anion to hydrogen peroxide, and CAT catalyzes then hydrogen peroxide into molecular oxygen and water. The effects of alcohol exposure on the activity or content of SOD and CAT are rather controversial in the literature, as the activity or content of SOD and CAT might increase, not change, or decrease, depending on the model, diet, amount, and time of alcohol feeding [13,24,28,29]. In this study, the activity of SOD was stimulated in the model with respect to the control group, but CAT decreased. Treatment with *Passiflora edulis* increased the content of CAT in alcoholic mice, which might protect the liver from the damage of alcohol. However, treatment with *Solanum muricatum* lowered the activity of SOD, which might be associated with increased liver injury.

### 2.5. Histopathological Evaluation

**Figure 5:**
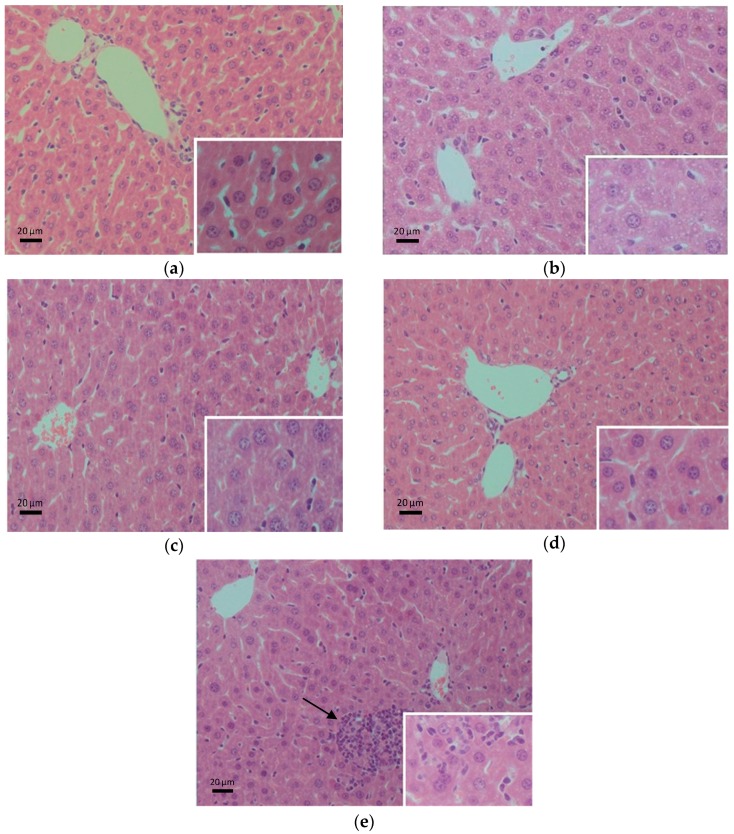
Representative liver sections stained with hematoxylin-eosin (H&E). Arrow indicates infiltration of inflammatory cells, which mainly occurred in the *Solanum muricatum* group. (**a**) histopathological image of liver in the control group; (**b**) histopathological image of liver in the model group; (**c**) histopathological image of the liver in the alcohol + *Syzygium samarangense* group; (**d**) histopathological image of the liver in the alcohol + *Passiflora edulis* group; (**e**) histopathology image of the liver in the alcohol + *Solanum muricatum* group.

A histopathological assessment of the liver was carried out in every group. As shown in Figure 5a, the liver of the control group mice displayed no pathological abnormalities (e.g., necrosis, inflammation, mononuclear cell aggregation, thrombus, or fatty degeneration). Figure 5b showed that alcohol induced extensive small fat droplets in the hepatocyte cytoplasm of the model group as compared to the control group. Livers of mice in the *Syzygium samarangense* (Figure 5c), *Passiflora edulis* (Figure 5d) and *Solanum muricatum* (Figure 5e) groups showed fewer small fat droplets in hepatocyte cytoplasm compared with the model group. However, liver of alcoholic mice treated with *Solanum muricatum* showed infiltration of inflammatory cells.

**Figure 6:**
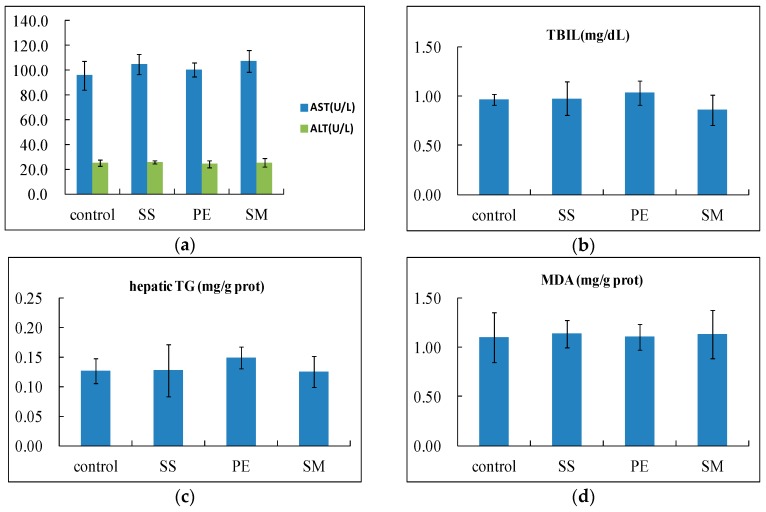
Effects of fruit juices alone (without alcohol) on the levels of ALT (**a**), AST (**a**) and TBIL (**b**) in serum as well as TG (**c**) and MDA (**d**) in the liver. SS: *Syzygium samarangense*; PE: *Passiflora edulis*; SM: *Solanum muricatum*.

The pathological changes observed in the alcohol-treated mice liver were consistent with the biochemistry parameters obtained. The administration of ethanol in mice resulted in mild steatosis histopathological changes. Reduction of histological damage was observed following the treatment of *Syzygium samarangense* and *Passiflora edulis*, confirming their hepatoprotective properties. Although the *Solanum muricatum* treatment group showed less microvesicular steatosis, inflammatory cellular infiltration was observed, which indicated that *Solanum muricatum* treatment can reduce hepatocyte steatosis, but deteriorate the inflammation induced by alcohol. On the other hand, fruit juices alone (without alcohol) did not alter the levels of ALT, AST, TBIL, ALP, TG or TP in the serum, nor the contents of hepatic TG or MDA, compared with the control group, and part of the results are shown in Figure 6, which shows that fruit juices (without alcohol) would not damage the liver. Thus, *Solanum muricatum* could not cause liver damage, but it could aggravate liver damage induced by alcohol.

To study liver damage caused by alcohol, different experimental models have been developed, such as the Lieber-DeCarli liquid diet model [30,31], and the chronic binge model [32,33]. Pair-feeding is adopted in the Lieber-DeCarli liquid diet model, which eliminates influence from factors other than ethanol. Thus, this model has been widely used. Pair-feeding can well be done in rats and baboons. On the other hand, the chronic binge model is also used in many studies [34,35]. The model does not need to adjust the diet composition and control the dietary intake. Thus, it can better simulate the human body alcohol intake, and can be used to evaluate the protective effect of natural products on liver injury induced by alcohol. However, this model lacks the pair-feeding, which would result in that stability of the model is not very well. In the present study, a 15-day alcohol gavage model (chronic binge model) was adopted, which increased the levels of AST, ALT, TBIL, TG in serum as well as of MDA and TG in the liver, and generated extensive small fat droplets in the hepatocyte cytoplasm. The change of these indicators showed that the liver injury model induced by 15-day alcohol gavage is practicable, and the model has also been adopted by other researchers [36,37].

The bioactive compounds and bioactivities of fruits, vegetables and medicinal plants mainly depend on their species. However, several factors could also affect the concentrations and proportions of bioactive compounds in these plants in different ways, such as different types of culture, sun exposure, rainfall, the degree of ripeness, and storage condition [38]. Thus, the fruits from different countries with different degrees of ripeness should be considered in the future study.

## 3. Materials and Methods

### 3.1. Chemicals and Reagents

SOD, MDA, GSH, CAT, and TG kits were purchased from Nanjing Jiancheng Bioengineering Institute (258-27 Central road, Nanjing, China). Other chemicals were of analytical grade.

### 3.2. Fruit Materials

The three fruits were obtained from markets in Guangzhou, China. The edible portion was separated by knife. Accurate weighing of a certain quality of the edible portion was mixed with water at a 1:5 solid-liquid ratio and ground into fine particles with a grinder. The sample was centrifuged at 5000× *g* for 10 min, and the supernatant was collected for animal study. Fresh fruit juices were prepared before every administration.

### 3.3. Animal Study

Male C57BL/6 mice weighing 20–25 g were used in this study. A total of 48 C57BL/6 mice were randomly divided into 8 groups with 6 mice in each group. They were maintained in a room with a controlled temperature of 22 ± 0.5 °C, 40%–60% relative humidity, and a 12-h light-dark cycle and were allowed free access to a basal pellet diet and tap water. The study was performed according to the “Principles of Laboratory Animal Care” and approved by the Institutional Animal Ethics Committee of School of Public Health, Sun Yat-sen University (1 March 2016, No. 2016-003). The alcohol + fruit-water mixture groups were treated daily with different fruit juice-water mixtures at the same dose (10 mL/kg body weight). A portion of 35% *v*/*v* ethanol at a dose of 3 g/kg body weight was used for the first 7 days, 40% *v*/*v* ethanol at a dose of 4 g/kg body weight was used for the following 7 days, and 52% *v*/*v* ethanol at a dose of 5 g/kg body weight was used on the 15th day [39]. For alcohol + fruit-water mixture groups, alcohol was firstly treated by intragastric administration, and fruit extract and water was then administered by gavage. In addition, fruit juice (without ethanol) groups were treated daily with different fruit juices (10 mL/kg) and distilled water. At the same time, the control group was given the same amount of distilled water for 15 days. Then, 9 h after the last oral administration, the animals were anesthetized, and blood samples were collected. The blood samples were centrifuged at 4000× *g* for 10 min to separate the serums. The serums were stored at –22 °C until the assay of ALT, AST, ALP, TP, TG, and TBIL. The liver was also taken from each animal. A piece of liver tissue was rapidly removed and fixed in 4% paraformaldehyde, and the rest of the liver was stored at −22 °C until use.

### 3.4. Measurement of Hepatic Injury in the Serum

The contents of ALT, AST, ALP, TBIL, TP and TG were measured by a Hitachi-7180 automated biochemistry analyzer (Hitachi, Tokyo, Japan) with a diagnostic reagent kit.

### 3.5. Measurement of MDA, SOD, GSH, CAT, and TG in the Liver

Liver tissue samples were homogenized in an ice-cold 0.9% NaCl solution. The homogenate (10%, *w*/*v*) was centrifuged at 4500× *g* for 10 min to obtain supernatant, which was used for biochemical analysis. The levels of MDA, SOD, GSH, CAT and TG in the liver tissue were determined by the commercial detection kits according to the manufacturer′s instructions.

### 3.6. Liver Histopathological Evaluation

A piece of liver was removed and fixed in 4% paraformaldehyde. Then, the liver was embedded and stained with hematoxylin-eosin in Google Biological Technology Co., LTD (Wuhan, China) for an assessment of histopathological alterations. Histopathological changes of stained livers were observed under a bright-field microscope.

### 3.7. Statistical Analysis

The results obtained were expressed as mean ± standard deviation (SD). Statistical significance was determined by a one-way analysis of variance (ANOVA) followed by a post-hoc least significant difference (LSD) test using SPSS 13.0 software (Armonk, New York, NY, USA). A value of *p* < 0.05 was considered statistically significant.

## 4. Conclusions

Treatment with *Syzygium samarangense* and *Passiflora edulis* can protect the liver from damages of alcohol, and the mechanism of the protective effect might be related to the enhancement of the antioxidant system. However, *Solanum muricatum* might aggravate alcohol-induced liver injury, and the mechanism of this effect needs to be further studied. Intake of *Syzygium samarangense* and *Passiflora edulis* with alcohol consumption can be recommended, but intake of *Solanum muricatum* with alcohol consumption is not advised. In addition, *Syzygium samarangense* and *Passiflora edulis* can also be further developed as a functional food or drug for the prevention and treatment of alcoholic liver disease.
